# The Association between Adverse Childhood Experiences, Adult Attachment-Related Characteristics, and Problematic Alcohol Use among Black American Emerging Adults

**DOI:** 10.1007/s40653-026-00934-y

**Published:** 2026-06-26

**Authors:** Diamonde C. McCollum, Nathan Meredith, Jenni B. Teeters

**Affiliations:** 1 Clinical-Community, Department of Psychology, University of South Carolina, Columbia, South Carolina USA; 2 Psychological Sciences Department, Western Kentucky University, Bowling Green, Kentucky USA

**Keywords:** ACEs, Adult attachment-related characteristics, Black Americans, Problematic alcohol use

## Abstract

Over 50% of individuals in the United States have had an Adverse Childhood Experience (ACE). ACE exposure has been associated with difficulties in emotion regulation, attachment-related insecurity, and increased risk for problematic alcohol use during emerging adulthood. Although prior research has linked ACEs, attachment-related characteristics, and problematic alcohol use, these relationships remain underexamined in Black American emerging adults, a population disproportionately exposed to ACEs. The present study examined associations among cumulative ACEs, adult attachment-related characteristics, and problematic alcohol use in a sample of Black American emerging adults. Participants were Black American emerging adults recruited via Prolific. Participants completed self-report measures of cumulative ACEs, adult attachment characteristics (closeness, dependency, and anxiety), and problematic alcohol use. Bivariate correlations and mediation analyses using PROCESS Model 4 were conducted to examine indirect associations between cumulative ACEs and problematic alcohol use through adult attachment subscales. Cumulative ACEs were positively associated with problematic alcohol use and attachment anxiety and negatively associated with attachment closeness and dependency. Significant indirect effects of cumulative ACEs on problematic alcohol use were observed through attachment dependency and anxiety subscales, but not for the closeness subscale. The results from the current study fill a gap in the literature surrounding ACEs, adult attachment-related characteristics, and problematic alcohol use within a sample of Black American emerging adults. Results highlight attachment-related anxiety and dependency as correlates of alcohol risk in the context of ACEs. Future longitudinal and intervention research is needed to further clarify these associations in Black American emerging adults.

In the United States, adverse childhood experiences (ACEs) are a rising concern with 51% − 62% of adults reporting at least one ACE and roughly 25% reporting three or more ACEs (Alvanzo et al., 2020; Merrick et al., 2018). ACEs are potentially traumatic events that occur prior to the age of 18, including physical, psychological, and sexual abuse, physical and emotional neglect, and household dysfunction (Crouch et al., 2019; Felitti et al., 1998). Household dysfunction includes parental separation or divorce, witnessing domestic violence, residing in a household with someone who engages in substance use or has a mental health disorder, and incarceration (Crouch et al., 2019; Felitti et al., 1998). ACEs also include community violence, peer victimization/isolation/rejection, and low socioeconomic status (Rutter, 2021). ACEs potentially influence adult attachment insecurity, which includes discomfort with intimacy and closeness with others and the belief that others are unreliable or untrustworthy (Mikulincer et al., 2003).

Research has shown that the more ACEs an individual experiences, the more likely they are to engage in substance use (Crouch et al., 2019). Emerging adults use substances more than any other age group, and heavy substance use among this age group contributes to a number of consequences, such as incarceration, developing a substance use disorder, and/or being involved in a car crash (Arnett, 2005; Crouch et al., 2019). Though previous research has identified connections between ACEs, adult attachment-related characteristics, and substance use in adult samples, little research has examined these relationships during emerging adulthood within marginalized populations (Murase et al., 2021; Perlman et al., 2016). Accordingly, the present study aims to examine associations between ACEs, adult attachment-related characteristics, and problematic alcohol use in a sample of Black American emerging adults.

ACEs have been associated with a maintained maladaptive stress response during childhood, which is linked to disruptions in emotional regulation, relationships, and physiological processes and may be associated with greater reliance on maladaptive coping behaviors, such as substance use, to temporarily relieve distress (Rogers et al., 2022). Research has also suggested that the presence of one ACE is significantly related to experiencing another ACE (Dong et al., 2004; Muller et al., 2012). Overall, higher ACE scores have been associated with greater feelings of negativity, instability, and perceptions of fewer life opportunities (Davis et al., 2018), suggesting these emotions may also be associated with increased vulnerability for maladaptive behaviors such as problematic alcohol use.

ACEs disproportionately impact Black Americans (Hampton-Anderson et al., 2021). While all demographic groups can experience ACEs, Black Americans report higher rates compared to Hispanic and White individuals (Hampton-Anderson et al., 2021). Research suggests that these disparities are partly associated with broader structural and contextual factors, including economic disadvantage (Bruner, 2017; Giovanelli et al., 2020). However, even with increased income, ACE exposure among Black Americans does not decrease at the same rate as observed in other demographic groups (Maguire-Jack et al., 2020), highlighting the persistence of disparities in childhood adversity.

ACEs have been associated with disruptions in the development of internal working models (IWMs), which may be linked to differences in an individual’s ability to form secure attachment-related characteristics in adulthood (Muller et al., 2012; Schmidt et al., 2020). The IWM shapes individuals’ self-image and expectations of care and stability in future relationships (Grady et al., 2017). Secure attachment in adulthood involves comfortability with intimacy and closeness with others (Hazan & Shaver, 2017; Mikulincer et al., 2003). Individuals with more securely attached characteristics are more likely to report adaptive emotion regulation and interpersonal functioning during times of stress and social adjustment (Mikulincer & Shaver, 2019), potentially protecting against risky behaviors (e.g., problematic substance use). Individuals who display characteristics of an insecure-anxious attachment may struggle with processing their emotions, and fear abandonment and rejection from others (Hazan & Shaver, 2017; Mikulincer et al., 2003). In contrast, insecure-avoidant attachment involves attempting to suppress negative feelings such as fear, anger, sadness, and shame (Mikulincer & Shaver, 2019). Both anxious and avoidant attachment orientations are characterized as forms of insecure attachment (Ainsworth, 2015; Muller et al., 2012), and are associated with difficulties in emotion regulation and reliance on maladaptive coping strategies, which may increase vulnerability to substance use during adulthood (Caspers et al., 2005; Kassel et al., 2007; Schindler et al., 2005).

Research indicates that ACEs in all categories are associated with a greater likelihood of having characteristics related to insecure attachment (Grady et al., 2017; Murphy et al., 2014), suggesting that all forms of ACEs can contribute to characteristics related to insecure attachment styles. Experiencing multiple ACEs may potentially be a risk factor for insecure attachment-related characteristics. Although research examining adult attachment characteristics within Black populations remains limited, available evidence suggests that early environmental stressors are meaningfully related to attachment-related outcomes in Black emerging adults (Montague et al., 2010). For example, research has shown that exposure to chronic stress during adolescence is associated with later physiological and psychological outcomes, and that attachment-related characteristics may be relevant for understanding variability in stress-related outcomes among Black individuals (Ehrlich et al., 2022). Collectively, the current body of work suggests that early adversity may shape IWMs and emotion regulation capacities in ways that increase vulnerability to insecure attachment-related characteristics in adulthood. In turn, these attachment-related processes may influence how individuals cope with distress, including the use of alcohol as a maladaptive regulation strategy. This conceptual framework positions adult attachment-related characteristics as a plausible pathway linking ACEs to problematic alcohol use in emerging adulthood.

In addition to findings that ACEs shape attachment-related emotion regulation processes, research has also linked ACEs to problematic substance use during emerging adulthood (Arnett, 2005; Marks et al., 2021; Rogers et al., 2022). Emerging adults have the highest rates of substance use of any age demographic (Marks et al., 2021). This age category also corresponds to elevated impulsivity, increased freedom and access to alcohol and drugs, as well as several transitions and stressors, such as entering college or the work force and leaving their caregivers home (Marks et al., 2021). A review of 28 studies examining alcohol outcomes found that ACE exposure was positively associated with increased alcohol use and problem drinking among emerging adults in 24 studies (Rogers et al., 2022). Shin and colleagues (2018) found that emerging adults who had multiple exposures to ACEs (in several categories) were at higher risk for problematic alcohol use compared to individuals who had not experienced any ACEs (Shin et al., 2018; Windle et al., 2018). These findings portray that experiencing single and cumulative ACEs is a risk factor for problematic alcohol use during emerging adulthood. One possible theory underlying the connection between ACEs and alcohol use is the self-medication hypothesis, which suggests that individuals use substances to numb, reduce, or escape from psychological pain (Broman et al., 2019; Khantzian, 1997). ACEs can have a long-term impact on cognitive and emotional deficits that can impede self-regulation, potentially increasing vulnerability for maladaptive coping behaviors such as substance use, to temporarily relieve distress (Marks et al., 2021; Rogers et al., 2022).

Research indicates that problematic alcohol use disproportionately affects minoritized populations due to cumulative ACEs. Lee and Chen (2017) found that Black Americans who experienced ACEs related to child abuse and household challenges were three times more likely to drink heavily than White and Hispanic individuals potentially due to ACEs in different categories (Lee & Chen, 2017), while Forster and colleagues (2018) found that Black Americans who reported alcohol use in the past 30 days had increased odds for alcohol use for every additional ACE they reported compared to White individuals (Forster et al., 2018). Building on the self-medication hypothesis, marginalized adults may self-medicate with substances to cope with the stressors related to ACEs, which may result in increased overall alcohol use (Marks et al., 2021). A systematic review found that 50% of studies examining ACEs and problematic alcohol use had primarily White samples, 22% were Hispanic, 16% were Asian, 6% were Black Americans, and 6% were multiethnic (Rogers et al., 2022), highlighting the need for more research in the Black community to analyze ACEs impact on risky health behaviors (e.g., alcohol use) during emerging adulthood.

Characteristics related to insecure attachment styles can potentially influence problematic substance use due to issues with emotion regulation and attempts to alleviate distress (Caspers et al., 2005). Individuals with qualities related to secure attachment styles may have more effective emotion regulation skills to process undesirable emotions compared to insecure attachment styles (avoidant and anxious), leading to use of ineffective methods to cope with undesirable emotions, such as risky substance use (Caspers et al., 2005; Kassel et al., 2007; Schindler et al., 2005). It was also found that individuals who had an alcohol use disorder were more likely to have characteristics related to insecure-avoidant or anxious attachment (Vungkhanching et al., 2004). Recently, Gidhagen and colleagues (2018) found that individuals with substance use disorders were more likely to have characteristics related to insecure attachment styles compared to nonclinical groups (Gidhagen et al., 2018). A systematic review with 37 original studies on attachment, problematic alcohol use, and other substance-related problems concluded that there is a link between insecure attachment-related characteristics and substance use, with elements of secure attachment being a protective factor against problematic alcohol use (Schindler, 2019). These findings suggest that qualities related to insecure attachment styles during adulthood are potentially a risk factor for problematic alcohol use.

Overall, little research has examined the connections between cumulative ACEs, adult attachment-related characteristics, and problematic alcohol use. Existing research, however, suggests that ACEs involving child maltreatment are associated with insecure-avoidant and anxious adult attachment characteristics and elevated risk for problematic substance use during emerging adulthood (Murase et al., 2021; Oshri et al., 2015; Pearlman et al., 2016). However, much of this literature has relied on predominantly White samples, limiting the generalizability of findings to other populations. As a result, additional research is needed to examine how cumulative ACE exposure, attachment-related characteristics, and problematic alcohol use are associated within historically underrepresented groups, including Black American emerging adults.

Despite growing evidence linking ACEs to both attachment-related characteristics and problematic alcohol use, several important gaps remain in the literature. First, much of the existing work has examined these variables separately, with relatively few studies considering ACEs, adult attachment-related characteristics, and alcohol-related outcomes together within the same model. Second, although emerging adulthood represents a developmental period marked by heightened vulnerability to both attachment-related difficulties and substance use, this group has received limited focused attention in prior research examining these associations. Third, existing studies have relied predominantly on White samples, leaving Black populations underrepresented in research examining ACEs, attachment-related characteristics, and alcohol use. Addressing these gaps is critical for advancing a more inclusive and developmentally appropriate understanding of how early adversity may be associated with later alcohol-related risk.

## Rationale for Current Study

There is little previous research on the connection between ACEs, adult attachment-related characteristics, and problematic alcohol use, specifically within Black American emerging adults. Accordingly, the present study aimed to examine associations between cumulative ACE exposure and problematic alcohol use within a sample of Black American emerging adults. Therefore, it is hypothesized that 1) cumulative ACEs will be positively associated with problematic alcohol use. It is also hypothesized that 2a) cumulative ACEs will be negatively associated with closeness and dependency adult attachment subscales, 2b) cumulative ACEs will be positively associated with the anxiety subscale of adult attachment, and 3) there will be an indirect effect of cumulative ACEs on problematic alcohol use through adult attachment scores on all three adult attachment subscales (closeness, dependency, and anxiety). Comprehending the associations between these variables could help improve intervention and prevention methods geared towards Black individuals who have experienced ACEs and engage in problematic alcohol use.

## Method

### Participants

Data for the present study were drawn from a larger study examining moderated mediation models of ACEs, attachment-related characteristics, and health risk behaviors. The current analyses focus exclusively on the subsample of Black American emerging adults in order to address gaps in the literature regarding ACEs, attachment-related characteristics, and problematic alcohol use within this population.

Participants were recruited from Prolific Academic, which is a national online survey platform. Black American males and females ages 18–35, who reside in the United States and speak/read English, were recruited for this study. Prolific compensated participants monetarily at a minimum of $8.00 per hour. There were 227 participants who had an average age of 24.3 years (*SD* = 3.14). Additionally, participants identified as 50.2% male, 47.6% female, and 60.4% heterosexual. All participants met inclusion criteria; thus, there were no participants excluded from the study. All procedures were approved by the Institutional Review Board of Westeen Kentucky University. After IRB approval, a survey was created on Qualtrics and was hosted on Prolific. Sufficient monetary funds were uploaded to the Prolific platform and compensated 227 participants at a minimum of $8.00 per hour for their time and participation.

### Procedure

Before engagement in the survey, individuals were presented with an informed consent form, which gave them the option to agree or disagree to participate in the survey. If they agreed to participate, they proceeded with the survey and responded to questionnaires related to demographics, ACEs, adult attachment-related characteristics, and problematic alcohol use. Due to the sensitive nature of ACEs and individuals engaging in alcohol use potentially under the legal drinking age limit, participants were reminded that their answers will remain anonymous and that they do not have to answer any questions that may create discomfort. Participants were also informed that they can withdraw from the study at any time, without any consequences. Participants answered attention check questions throughout the survey to assess attentiveness. At the end of the survey, individuals were presented with a debriefing document explaining the purpose of the study, lab contact information, and information about available mental health resources should participation result in distress.

### Measures

*Adverse Childhood Experiences*. Adverse Childhood Experiences Scale-International Questionnaire (WHO, 2012) is a 36-item scale that predicts long-term health disparities due to traumatic events experienced directly or witnessed during childhood. The ACES-IQ can be analyzed in two different ways, (1) in a binary format, answering “yes” or “no” to an item like, “Did a parent, guardian or other household member spank, slap, kick, punch or beat you up?” or (2) items can be analyzed in a frequency format by answering “refused,” “never,” “many times,” “a few times,” and “once” to an item like “Did you see or hear a parent or household member in your home being slapped, kicked, punched or beaten up?” For this study, the ACES-IQ was analyzed in a frequency format, and summed to create a continuous score, with higher values reflecting greater cumulative ACE exposure. Consistent with World Health Organization (WHO) guidelines, this approach allows for assessment of both the presence and severity of ACEs. Frequency scoring captures variability in exposure intensity and distinguishes between single and repeated experiences, in turn providing a more nuanced representation of cumulative adversity than binary scoring, which treats any exposure as equivalent (WHO, 2012). Internal consistency for the current study was good (α = 0.80).

*Adult Attachment Styles.* Adult attachment-related characteristics were measured using the Revised Adult Attachment Scale- Close Relationships (RAAS-CR; Collins, 1996). This questionnaire included 18 questions that assessed attachment-related characteristics across close interpersonal relationships, including family members, romantic partners, and close friends. The RAAS-CR measures three dimensions of attachment including closeness (how comfortable an individual feels with emotional closeness), dependency (one’s ability to depend on another person), and anxiety (fear of being abandoned or rejected). This questionnaire uses a five-point Likert scale and individuals respond based on how they generally feel in relationships with family members, romantic partners, and close friends, with response options ranging from “not at all characteristic of me” to “very characteristic of me”. There are six questions related to closeness, six questions related to dependency, and six questions related to anxiety in close relationships (Collins, 1996). An example of a closeness statement would be “I find it relatively easy to get close to people.”, a dependency statement would be “I find it difficult to allow myself to depend on others.”, and an anxiety statement would be “I often worry that other people don’t really love me.” The total score on each subscale (closeness, dependency, anxiety) was calculated by summing items within each domain. Scoring higher on the attachment anxiety subscale would indicate greater fears of abandonment and rejection, while lower scores on closeness and dependency would reflect lower comfortability with closeness within relationships and reduced ability to depend on others. Thus, higher scores on attachment anxiety and lower scores on attachment dependency and closeness relate to insecure adult attachment styles, while secure adult attachment styles would be associated with higher scores on closeness and dependency subscales and lower attachment anxiety. The present study used the RAAS-CR subscales as continuous dimensional measures of attachment-related processes, rather than categorical attachment style classifications. This approach is especially appropriate given concerns regarding the cultural validity of categorical attachment classification in diverse samples. Although the RAAS-CR has been widely used in adult populations, its development and validation have largely relied on predominantly White samples. Therefore, findings should be interpreted as reflecting attachment-related characteristics rather than fixed attachment styles. The subscales had good internal consistency in the current study; closeness subscale (α = 0.78), anxiety subscale (α = 0.86), and dependency subscale (α = 0.80).

*Problematic Alcohol Use.* Problematic alcohol use will be measured using the Alcohol Use Disorders Identification Test (AUDIT; Saunders et al., 1993). This questionnaire was developed by the World Health Organization, including 10 self-report item questions, with the purpose of identifying individuals who engage in alcohol use that is harmful to their health (Saunders et al., 1993). There are questions related to alcohol consumption frequency, drinking behaviors, and problems that are caused by alcohol and includes a nominal response format. An example of a frequency question would include, “How many standard drinks containing alcohol do you have on a typical day when drinking?” with answers “1 or 2” “3 or 4,” “5 or 6,” “7, 8, or 9” or “10 or more”(Saunders et al., 1993). An example of alcohol-related problems would be “Have you or someone else been injured because of your drinking?”, with response options “no”, “yes, but not in the last year”, and “yes, during the last year.” A score of 8 or more indicates harmful drinking (Saunders et al., 1993). Consistent with WHO guidance, AUDIT total scores can be interpreted using established cutoffs (e.g., scores of 1–7 indicating low-risk consumption, 8–15 indicating hazardous or harmful alcohol use, and 16–19 indicating harmful use, and greater than 20 suggesting probable alcohol dependence; Babor et al., 2001). However, in the present study, the AUDIT was analyzed as a continuous measure to retain variability in alcohol-related risk and avoid loss of information associated with categorical classification (Dawson et al., 2010). Accordingly, higher scores reflect greater problematic alcohol use, with scores ranging from 0 to 40 (Saunders et al., 1993). The AUDIT demonstrated high internal consistency in the current sample (α = 0.86).

### Data Analysis Plan

To test the first and second hypotheses (1: ACEs will be positively associated with problematic alcohol use, 2a: ACEs will negatively be associated with closeness and dependency adult attachment subscales, and 2b: ACEs will be positively associated with the anxiety subscale of adult attachment), bivariate correlations were conducted. This allowed for examination of whether ACEs are correlated with adult attachment subscales and problematic alcohol use in Black American emerging adults.

To investigate the third hypothesis (that adult attachment-related characteristics would account for indirect associations between cumulative ACE exposure and problematic alcohol use), a mediation analysis was performed using PROCESS model 4. In the mediation model, the ACES-IQ total score was the predictor variable, while total scores on closeness, dependency, and anxiety subscales were mediating variables, and total AUDIT scores as the outcome variable, representing problematic alcohol use. This approach is consistent with contemporary mediation frameworks, which do not require a significant total effect between predictor and outcome variables in order to estimate indirect effects (Hayes, 2013; Zhao et al., 2010).

Age, gender, and educational attainment were included as covariates in all regression and mediation models to account for the potential influence on attachment subscales and problematic alcohol use. Prior to hypothesis testing, the following assumptions were evaluated. Linearity and normality of residuals were assessed via examination of residual plots. Multicollinearity was assessed using variance inflation factors (VIFs), with values below commonly accepted thresholds indicating no evidence of multicollinearity. Outliers were examined using standardized residuals. No violations of assumptions were observed.

## Results

### Descriptive Statistics

There were 227 participants who completed the ACE-IQ, which includes 13 subcategories (emotional abuse, physical abuse, sexual abuse, emotional neglect, physical neglect, household problem alcohol and drug use, incarcerated household member, residing with someone who has a mental illness, domestic violence, parental loss/separation, bullying, community violence, and collective violence). All findings for the sample are reported in supplementary tables. Of these participants, 100% reported at least one ACE. The highest reported subgroup within the ACE-IQ was emotional neglect, with bullying, and emotional abuse following. Subgroups of collective violence, sexual abuse, household incarceration, and household problem alcohol and drug use were the least reported ACE-IQ subcategories. For the AUDIT total score, the majority of participants were low risk for problematic alcohol use.

### Hypothesis Testing

Bivariate correlations were examined to assess associations between ACEs, adult attachment-related characteristics, and problematic alcohol use. ACEs were significantly positively correlated with problematic alcohol use (*r* = .292, *p* < .001). ACEs were also significantly positively associated with anxiety attachment (*r* = .328, *p* < .001) and significantly negatively associated with closeness (*r* = − .173, *p* = .009) and dependency (*r* = − .216, *p* = .001) attachment subscales. Among the attachment subscales, only dependency was significantly correlated with problematic alcohol use (*r* = .132, *p* = .047); closeness and anxiety were not (see Table 1).

**Table 1:** Bivariate correlations between ACEs-IQ, adult attachment subscales, and problematic alcohol use (N = 227)

Variable	M	SD	1	2	3	4	5
1. ACE-IQ (sum score)	19.69	11.37	—				
2. Attachment Anxiety	18.38	6.14	0.33**	—			
3. Attachment Closeness	17.92	5.19	− 0.17**	− 0.44**	—		
4. Attachment Dependency	15.91	5.11	− 0.22**	− 0.55**	0.61**	—	
5. Problematic Alcohol Use (AUDIT)	3.46	4.94	0.29**	0.06	0.11	0.13*	—

Closeness, dependency, and anxiety are subscales of the RAAS-CR adult attachment scale

*p < .05. **p < .001

**Fig. 1:**
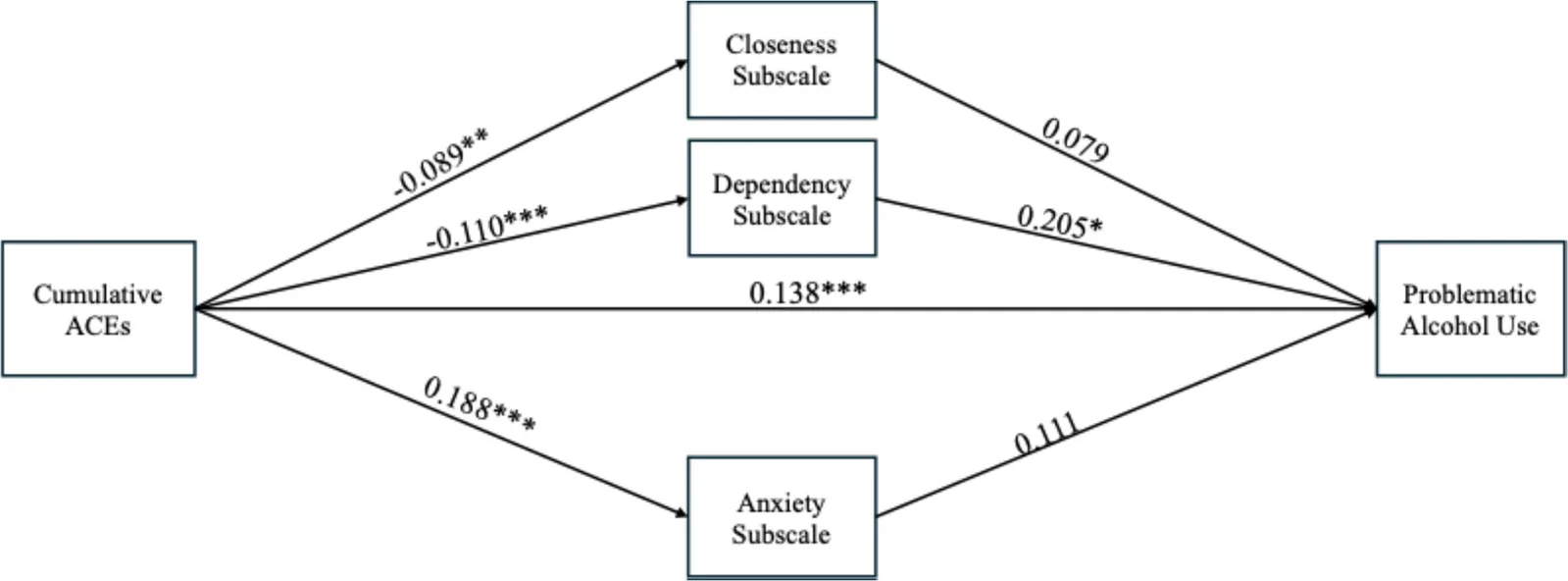
Indirect effect of closeness, anxiety, and dependency attachment-related characteristics between ACEs and problematic alcohol use

A mediation analysis was completed using PROCESS model 4 to examine whether there was an indirect effect of ACES on problematic alcohol use through the adult attachment subscales (closeness, dependency, and anxiety; See Table 1). A model was examined with cumulative ACEs as the predictor variable, closeness, dependency, and anxiety adult attachment subscales as parallel mediators, and problematic alcohol use as the outcome variable (See Fig. 1). ACEs were significantly negatively associated with closeness (*b* = − 0.089, SE = 0.031, *p* = .01) and dependency (*b* = − 0.110, SE = 0.031, *p* < .001), and significantly positively associated with anxiety (*b* = 0.188, SE = 0.035, *p* < .001). Only attachment dependency was significantly associated with problematic alcohol use in the model (*b* = 0.205, SE = 0.083, *p* = .015); closeness and anxiety were not significant predictors. The indirect effects of ACEs on problematic alcohol use were significant through dependency (effect = − 0.023, 95% CI [-0.050, − 0.005]) and anxiety (effect = 0.021, 95% CI [0.001, 0.043]), but not through closeness (effect = − 0.007, 95% CI [-0.026, 0.006]; See Table 3). The total indirect effect was not significant (effect = − 0.009, 95% CI [-0.038, 0.014]). The direct effect of ACEs on problematic alcohol use remained significant (*b* = 0.138, SE = 0.030, *p* < .001; See Table 3). These findings suggest partial mediation by dependency and anxiety attachment subscales in the present sample. Age, gender, and education were included as covariates in all models.

**Table 2:** Indirect Analysis Predicting Problematic Alcohol Use (*N* = 227)

Path	Predictor → Outcome	b	SE	t	*p*	95% CI
**a₁**	ACEs → Closeness	− 0.089	0.031	-2.86	0.005**	[-0.149, − 0.028]
**a₂**	ACEs → Dependency	− 0.110	0.031	-3.62	< 0.001***	[-0.170, − 0.050]
**a₃**	ACEs → Anxiety	0.188	0.035	5.38	< 0.001***	[0.119, 0.257]
**b₁**	Closeness → AUDIT	0.079	0.077	1.03	0.306	[-0.073, 0.232]
**b₂**	Dependency → AUDIT	0.205	0.083	2.46	0.015*	[0.041, 0.369]
**b₃**	Anxiety → AUDIT	0.111	0.065	1.71	0.089	[-0.017, 0.238]
**c′**	ACEs → AUDIT (direct effect)	0.138	0.030	4.61	< 0.001***	[0.079, 0.198]

*p < .05. **p < .01.***p < .001

**Table 3:** Indirect effects (Bootstrapped, 5,000 Samples)

Mediators	Indirect Effect	Boot SE	95% Boot CI
Closeness	-.007	.008	[-.026, .006]
Dependency	-.023	.011	[-.050, -.005]
Anxiety	.021	.011	[.001, .043]
Total Indirect Effect	-.009	.013	[-.038, .014]

## Discussion

The primary goals of this study were to examine the connection between adverse childhood experiences, adult attachment-related characteristics, and problematic alcohol use among Black American emerging adults. In addition to highlighting this association, the second aim was to investigate whether ACEs and problematic alcohol use were indirectly related through adult attachment subscales (closeness, dependency, and anxiety).

### Hypothesis 1

Was supported in that cumulative ACEs were positively associated with problematic alcohol use, consistent with a previous literature review finding 24 studies associating ACE exposure with increased alcohol use and problematic drinking among emerging adults (Rogers et al., 2022). Moreover, previous studies have emphasized that cumulative ACEs and/or ACEs in multiple subcategories were associated with problematic alcohol use (Forster et al., 2023; Shin et al., 2018; Windle et al., 2018). These findings indicate a robust association between cumulative ACE exposure and problematic alcohol use during emerging adulthood. Hypothesis 2a was supported because closeness and dependency adult attachment subscales were significantly negatively associated with ACEs. These findings are consistent with prior work linking childhood adversity to attachment-related insecurity and support the current findings of negative associations between cumulative ACE exposure and closeness (how comfortable an individual feels with closeness) and dependency (one’s ability to depend on another person) adult attachment subscales.

### Hypothesis 2b

Was supported because the anxiety adult attachment subscale was significantly positively associated with ACEs. Research supports the current findings of the increased association of the anxiety (fear of being abandoned or rejected) adult attachment subscale to cumulative ACEs (Riggs & Kaminski, 2010; Unger & De Luca, 2014). These findings are consistent with theoretical perspectives suggesting that childhood adversity may result in increased fears of abandonment and rejection, which in turn may threaten one’s ability to form characteristics related to secure attachment styles. Prior research has placed an emphasis on ACEs related to child maltreatment, threatening one’s ability to form secure adult attachment styles; however, the current study conceptualizes cumulative ACEs across a wide range of categories as a potential risk factor for attachment-related insecurity.

The third hypothesis was partially supported in that the indirect effect of cumulative ACEs on problematic alcohol use through adult attachment subscales dependency and anxiety, but not closeness, were significant. Prior research suggests that ACE exposure is associated with cognitive and emotional deficits that may be relevant to the development of characteristics related to attachment-related insecurity (Rogers et al., 2022). Other studies have also found that ACEs related to childhood maltreatment are associated with characteristics related to insecure adult attachment (Riggs & Kaminski, 2010; Unger & De Luca, 2014). Research has also indicated that ACEs in all categories are associated with a greater likelihood of having characteristics related to insecure attachment (Grady et al., 2017; Murphy et al., 2014). Lastly, it was found that women who experienced intimate partner violence, with higher scores on the attachment anxiety subscale and lower scores on the attachment closeness subscale, had significantly higher self-reported PTSD symptoms (Woodward et al., 2013). These findings portray the need to increase and encourage development of characteristics related to secure attachment (e.g., adaptive emotion regulation, trustworthiness), to buffer against the damaging effects of traumatic events. Collectively, these findings suggest that attachment-related characteristics may be relevant targets for future prevention and intervention efforts aimed at reducing alcohol-related risk among Black emerging adults with histories of childhood adversity, while still acknowledging that the present findings do not permit causal conclusions.

Additionally, although limited, research has supported that characteristics related to insecure adult attachment styles mediate the relationship between ACEs and substance use (Murase et al., 2021; Perlman et al., 2016). Pearlman and colleagues (2016) found that characteristics of avoidant and anxious attachment styles indirectly impacted the relationship between emotional abuse and coping via substance use. In further support, Murase and colleagues (2021) found that individuals who had characteristics related to insecure-avoidant attachment during adulthood experienced more problematic alcohol use due to severe physical abuse during childhood. This suggests that characteristics related to insecure adult attachment styles should be considered as a potential risk factor to problematic alcohol use. Lastly, the indirect effect of cumulative ACEs on problematic alcohol use was not significant through the closeness adult attachment subscale; this is surprising given prior research suggesting that characteristics related to secure adult attachment styles, such as closeness with others, are a protective factor for problematic alcohol use (Gidhagen et al., 2018; Schindler, 2019).

Several factors may explain this unexpected null finding. First, there may be a measurement mismatch. The RAAS-CR closeness subscale measures comfort with emotional intimacy in close relationships, which may not directly correspond with the specific coping mechanisms related to alcohol use. In contrast, the dependency and anxiety subscales may better capture interpersonal vulnerabilities that align with problematic alcohol use as means to self-medicate. Second, the RAAS-CR was developed and validated primarily in White samples, which raises questions about its cultural appropriateness for measuring attachment characteristics in Black American emerging adults. Third, there was low variability in total AUDIT scores, so the predominantly low-risk drinking pattern observed in this sample may have created a floor effect, limiting our ability to detect protective effects. Finally, the study may have been underpowered to detect small effects between certain attachment dimensions and problematic alcohol use.

Furthermore, the significant indirect effect of cumulative ACEs on problematic alcohol use through attachment dependency and anxiety subscales suggests that ACEs may be associated with greater attachment anxiety and lower ability to depend on others in emerging adulthood, which may co-occur with more problematic patterns of alcohol use. Though we did not examine coping motives as part of our model, previous work has suggested individuals with trauma exposure may use alcohol to cope with trauma-related distress (Broman et al., 2019; Khantzian, 1997). These findings underscore the importance of considering attachment-related factors when examining alcohol use patterns among individuals with higher levels of childhood adversity. Prior research has documented disproportionate exposure to ACEs among Black Americans (Merrick et al., 2018; Hampton-Anderson et al., 2021). These broader contextual factors, including racial discrimination, systemic oppression, and inequality, are important to consider in understanding patterns of childhood adversity in this population (Hampton-Anderson et al., 2021; Maguire-Jack et al., 2020). However, the current study did not assess discrimination, racism, or related sociocultural variables and therefore cannot appropriately speculate their role in the observed associations. In theory, reducing ACEs may provide increased opportunities to develop secure adult attachment-related characteristics and could potentially reduce problematic alcohol use during emerging adulthood. Characteristics related to secure adult attachment styles have been shown to be an adaptive resilience resource in times of need and social adjustment (Mikulincer & Shaver, 2019). Accordingly, interventions and resources that support the development of secure attachment-related characteristics during emerging adulthood may be beneficial for promoting emotion regulation capacities and adaptive coping among individuals with histories of cumulative adversity. More research examining these research questions in larger samples of Black Americans is needed as this population has been historically excluded from research in this area.

### Limitations, Implications, and Conclusions

Overall, the findings within this study are noteworthy and provide a unique perspective on the association between cumulative ACEs, adult attachment-related characteristics, and problematic alcohol use, but it is important to consider several limitations. Due to the cross-sectional nature of this study, causal inferences regarding the relationships between ACEs, adult attachment-related characteristics, and problematic alcohol use cannot be established. Longitudinal studies measuring ACEs, adult attachment-related characteristics, and substance use during adolescence and emerging adulthood are needed to better examine the initiation of alcohol use and potential problematic alcohol use during emerging adulthood and beyond. Additionally, all measures required retrospective reporting of behaviors which may be subject to recall biases. It is possible that participants may have underreported certain experiences or behaviors due to social desirability concerns or varying interpretations of questions. Specifically, for problematic alcohol use, future research should use more objective measures to examine alcohol use, such as ecological momentary assessment or daily diaries, to get more precise responses regarding daily alcohol use.

Moreover, due to using the Adverse Childhood Experiences- International Questionnaire, which has 13 subcategories of ACEs compared to the original Adverse Childhood Experience Questionnaire (3 subcategories), it is difficult to compare our findings to previous studies. However, this can also be seen as a strength because the present study included a much more diverse set of ACEs. Further, the current study used the RAAS-CR to measure continuous subscales related to adult attachment styles in Black populations, which is a significant contribution to the literature. However, due to the lack of research with attachment styles in Black populations, it remains unclear if the RAAS-CR is an appropriate measurement for Black communities, as its development and validation were conducted primarily in White samples. Future research using continuous adult attachment scales should be used in diverse populations to determine if modifications are needed for the questionnaire and to ensure cultural sensitivity. Lastly, the current study recruited participants through Prolific Academic, which provided an opportunity to have a diverse sample; however, potential self-selection bias should be considered, as findings may not generalize to individuals who do not participate in online research and/or those who are unaware of Prolific Academic. Future research should include individuals from online and within the community to measure ACEs, adult attachment-related characteristics, and problematic alcohol use for greater generalizability.

Despite the limitations, the present study adds important findings to the literature on ACEs, adult attachment-related characteristics, and problematic alcohol use in Black American emerging adults. This study used Prolific Academic, which has been shown to have a more diverse participant pool compared to Amazon Mechanical Turk (another popular online survey platform). The current sample was unique with 100% of individuals selecting Black/African American, 50.2% male and 47.6% female, increasing generalizability. To our knowledge, this is the first study current to examine cumulative ACEs, adult attachment-related characteristics, and problematic alcohol use within a sample of Black American emerging adults. Prior studies have focused on ACEs related to child maltreatment (physical, sexual, and emotional abuse), characteristics related to insecure adult attachment styles, and problematic alcohol use in majority White populations.

This study provides several interesting avenues for future research. It would be interesting to examine how these constructs relate to other risk behaviors that commonly co-occur with alcohol use in emerging adulthood, such as sexual behavior, suicidal behavior, and co-use of alcohol and cannabis. Additionally, examining the subcategories from the ACES-IQ and whether or not certain categories are more strongly connected to problematic alcohol use would be worthwhile. Given that dependency and anxiety subscales accounted for associations between ACEs and problematic alcohol use in the present study, future research should examine interventions that specifically target these attachment-related characteristics, such as interpersonal psychotherapy (IPT; Althobaiti et al., 2020), and Dialectical Behavioral Therapy (DBT). This is especially important as most ACEs result from interpersonal relationships. IPT, which emphasizes interpersonal functioning and emotion regulation, two processes closely related to the relevant attachment characteristics identified in the present study, may be especially relevant for individuals with histories of childhood adversity. This relevance is underscored by the current findings, which suggest that individuals with ACEs who report lower ability to depend on others and higher attachment anxiety may be at elevated risk for problematic alcohol use. Research has supported that individuals who have PTSD symptoms that receive IPT have better interpersonal skills, due to being taught skills related to effective emotion regulation (Althobaiti et al., 2020). Although present findings do not support causal conclusions, interventions such as IPT could serve to buffer the effects of ACEs and encourage development of characteristics related to secure adult attachment styles, thus reducing engagement in problematic alcohol use during emerging adulthood. However, this approach should be further examined in large, diverse populations and longitudinal research.

Further, teaching Dialectical Behavioral Therapy (DBT) interpersonal effectiveness skills within populations exposed to traumatic experiences may encourage development of secure attachment-related characteristics, due to individuals learning how to cope with stressors associated with interpersonal relationships. Such skills may be particularly important given the lower ability to depend on others and higher attachment anxiety. Research has shown that women who had PTSD symptoms related to childhood sexual abuse had decreased PTSD symptoms as well as improvements in social functioning, acceptance, and trauma-related emotions after receiving DBT (Choi-Kain et al., 2021). Although these findings are speculative and the present study’s cross-sectional design prevents causal interpretation, DBT’s emphasis on interpersonal effectiveness and emotion regulation may address the specific attachment related vulnerabilities identified as relevant in the present study. However, future intervention research utilizing longitudinal designs and diverse samples is necessary to determine whether DBT can effectively target these pathways and reduce problematic alcohol use among emerging adults with histories of ACEs.

The current study provides valuable information concerning the associations between cumulative ACEs, adult attachment-related characteristics, and problematic alcohol use in a sample of Black American emerging adults. Notably, results showed that the indirect effect of cumulative ACEs on problematic alcohol use through adult attachment dependency and anxiety subscales, providing evidence of indirect associations between ACEs and problematic alcohol use via these attachment-related characteristics. Moreover, given that prior research has documented that Black Americans are more likely to experience ACEs (Hampton-Anderson et al., 2021), future research should examine whether structural interventions addressing discrimination and systemic oppression are associated with changes in the development of insecure adult attachment-related characteristics, and problematic alcohol use. Given that this is among the first studies to examine these constructs and that problematic substance use peaks during emerging adulthood (Marks et al., 2021), future studies are needed to decipher if these findings replicate in other diverse populations (e.g., other race/ethnicities, other age groups, etc.).

## Data Availability

The data that support the findings of this study are not publicly available due to sensitivity purposes but are available from the corresponding author upon reasonable request.
